# Gene Therapy: A Potential Approach for Cancer Pain

**DOI:** 10.1155/2011/987597

**Published:** 2011-06-09

**Authors:** Chalonda R. Handy, Christina Krudy, Nicholas Boulis

**Affiliations:** Department of Neurosurgery, Emory University, 101 Woodruff Circle, Rm 6339, Atlanta, GA 30322, USA

## Abstract

Chronic pain is experienced by as many as 90% of cancer patients at some point during the disease. This pain can be directly cancer related or arise from a sensory neuropathy related to chemotherapy. Major pharmacological agents used to treat cancer pain often lack anatomical specificity and can have off-target effects that create new sources of suffering. These concerns establish a need for improved cancer pain management. Gene therapy is emerging as an exciting prospect. This paper discusses the potential for viral vector-based treatment of cancer pain. It describes studies involving vector delivery of transgenes to laboratory pain models to modulate the nociceptive cascade. It also discusses clinical investigations aimed at regulating pain in cancer patients. Considering the prevalence of pain among cancer patients and the growing potential of gene therapy, these studies could set the stage for a new class of medicines that selectively disrupt nociceptive signaling with limited off-target effects.

## 1. Introduction

The inability to control chronic pain remains one of the most significant clinical problems facing the medical establishment. Although the neuroanatomy of pain is relatively well understood, meaningful modulation of nociceptive pathways has not been established. Pain persists in association with a number of disorders, and its origin is not always apparent. In fact, it is one of the symptoms experienced by as many as 90% of cancer patients at some point during the course of the disease [1, 2]. This high incidence of pain among cancer sufferers may be due to the scarcity of elaborate studies directed towards reducing pain specific to the cancer patient. 

The study of these pain syndromes has traditionally employed empirical techniques that investigate the pathomechanisms of pain in isolation from tumorigenesis. Pain experienced by cancer patients is often treated as a separate entity from the cancer itself. Nevertheless, the undermanagement of pain can have drastic effects on survival and overall quality of life [3, 4]. Startling reports indicate that 46% of dying patients lack adequate pain treatment at the time of death [3, 4]. These observations beg the need for improved methods for cancer pain control. Furthermore, many commonly used pharmacological approaches to cancer pain lack anatomical specificity and often result in off-target and dose-limiting side effects that prevent lasting relief and create new sources of suffering. Similarly, surgical approaches to pain lack functional specificity, affecting multiple neural systems in a given anatomical target. 

In contrast, gene therapy may provide a means to overcome these barriers of specificity in the treatment of cancer pain. Moreover, in the scope of chronic pain syndromes, oncological pain may prove to be a suitable initial target for clinical translation. The terminal nature of many types of cancer limits the potential risk of radical approaches, while the severity of the pain syndromes justifies the need for extreme innovation. Substantial advancements have been made in viral vector design to establish gene therapy as a viable approach for pain management. In addition, the success of a variety of Phase I trials at demonstrating safety in gene therapy has significantly reduced safety concerns. 

This paper will provide a rationale for the development of new approaches to cancer pain management by making an argument for the use of viral vector-based approaches to modulate the nociceptive cascade. By evaluating established approaches for pain management and preclinical studies that determine the benefits of gene therapy for chronic pain this paper will make an argument for the translation of these methods to the cancer patient. It is important to note that the study of gene-based pain control is not limited to viral vector-derived strategies. There are a number of gene therapy approaches that have been used to investigate chronic pain (i.e., antisense oligonucleotides, gene silencing methods, etc.). However, this paper is limited to the scope of viral vector delivery of transgenes that have been shown to result in an analgesic effect. The pathways of nociception are not summarized to reduce overlap with a number of other reviews that effectively describe molecular and cellular mechanisms that underlie pain transmission [5–7]. 

The goal of this paper is to provide a comprehensive snapshot of laboratory investigations that demonstrate the benefits of viral vector delivery of therapeutic genes as well as highlight human studies that are aimed at determining the safety and feasibility of gene therapy for the regulation of pain in cancer patients. Furthermore, since the field of gene therapy has evolved to include cell therapy (*ex vivo* gene therapy), cellular-based approaches for pain attenuation are also described. It is important to note that most of the studies described in this paper were not conducted in an attempt to modulate pain specific to tumorigenesis. However, a number of factors shown to be responsible for the generation and maintenance of pain appear to also be involved in cancer-related nociception, suggesting an overlap of pain signaling pathways [8, 9]. Therefore, it is reasonable to assume that therapies shown to be effective in the absence of tumorigenesis may offer some degree of benefit to cancer pain. The substantial advancements that have been made in viral vector technology and our increased knowledge of neuronal circuits involved in the transmission and modulation of somatosensory information have increased enthusiasm for the potential success of gene and cell therapies for cancer-related pain. Hence, gene therapy-based pain management could prove to be a valuable interventional procedure that sets the stage for a new class of medicines effective at alleviating pain symptoms characteristic of various etiologies and multiple levels of severity.

## 2. Types of Cancer Pain

Cancer pain can be either nociceptive, neuropathic, or both. Nociceptive pain can be further distinguished as being somatic or visceral. Somatic cancer pain usually occurs secondary to bone metastasis. It can also be due to pathological fractures, surgery, or cancer treatment. It usually involves the activation of inflammatory mediators in that tumor and humoral factors such as prostaglandins, cytokines, and growth factors can act locally to stimulate nociceptors. Nonsteroid anti-inflammatory agents (NSAIDs) are commonly used to treat somatic pain either alone or in combination with opiate analgesics. 

Visceral cancer pain is usually poorly localized and is sometimes referred to cutaneous points in nonvisceral areas such as muscle or skin. This pain may not necessarily be related to tissue injury, but can be evoked due to distension, inflammation, irritation of mucosal surfaces, or impaction. Hence a tumor may go undetected unless it gets large enough to trigger certain factors to excite normally “silent” visceral nociceptive sensory receptors that respond to mechanical stimuli. This information is then relayed to sympathetic and parasympathetic efferent fibers that terminate in the spinal cord. The referred pain associated with visceral nociception is believed to be the consequence of the dual innervation or convergence of somatic and visceral structures by common fibers. Like somatic pain, combinations of opioids and NSAIDS are usually used to manage visceral cancer pain.

Neuropathic cancer pain results from damage to the peripheral (PNS) and central nervous system (CNS). During conditions of inflammation or neuropathy, primary afferents can become sensitized resulting in an amplification of signaling and modifications in neuronal response. These changes can cause nonnoxious stimuli to be perceived as painful (allodynia) or result in an exaggerated response to stimuli that is normally painful (hyperalgesia). In addition, denervation may result in spontaneous activity in central nociceptive neurons both at the spinal and thalamic levels.

Neuropathic pain is common in cancer and can be the result of tumor nerve compression or a side effect of chemotherapy, radiation, or surgery. Cancer-related neuropathic pain has been investigated using rodent or canine models [10–18]. In most cases, bone cancer pain was achieved by injections of tumor cells into the intramedullary space of the tibia or femur [10, 11, 15, 16]. Nociceptive behaviors indicative of pain such as vocalizing, guarding of the affected limb, differences in weight bearing between hind paws, mechanical allodynia, and/or hyperalgesia as well as neurochemical changes in the spinal cord are commonly assessed. Additionally, animals with naturally occurring tumors have also been utilized to evaluate cancer-related neuropathic pain states such as the spontaneous osteosarcoma canine model and models of pancreatic cancer, benign neuroma, and squamous cell carcinoma [12–14]. 

Neuropathic pain in the cancer patient often results from cancer treatment. In fact, the drugs vincristine, paclitaxel, and cisplatin often yield neuropathic pain [15]. To understand therapy-related changes that result in central sensitization, chemotherapy-related pain models have been developed in rodents. These animals studies involve either the intravenous administration of vincristine or the intraperitoneal injection of paclitaxel or cisplatin [15]. Like in human conditions, hypomyelination and degeneration of dorsal roots can be observed indicating the severity of the morphological changes induced by drug administration. 

Sodium channel blockers are commonly used to treat neuropathic cancer pain. The drawback of this therapy is that these agents are not selective enough and can result in undesirable effects on the CNS and cardiovascular systems. Therefore, the modulation of neurotransmitter activity by the use of glutamate receptor antagonists (ketamine and amantadine) or gamma-amino butyric acid (GABA) analogs (gabapentin) can also be used to reduce spontaneous firing in nociceptive pathways and the dorsal horn, helping to restore inhibitory control in the CNS [19–21]. 

Opiates are generally the last class of drugs to be used for neuropathic cancer pain. Unfortunately, neuropathic pain generally responds poorly to opiate therapy, and considerable side effects are often associated with the resulting need for high opiate doses [19, 22]. However, the coupling of opiate-associated peptides with viral vectors for targeted opiate delivery has been effective in achieving meaningful outcomes absent in adverse reaction. Although few studies have explored the advantages of gene therapy-based approaches to treat neuropathic cancer pain, there are, in fact, human trials being conducted to evaluate the benefits of gene therapy for cancer pain. These investigations were based on preclinical studies that demonstrated the analgesic effects of viral vector-driven proenkephalin expression for the attenuation of neuropathic pain in a model of bone cancer pain [23, 24]. These studies offer a rationale for the use of gene therapy to treat pain associated with cancer-related conditions. By establishing models to monitor the pathomechanisms and behavioral phenotypes associated with the generation and maintenance of cancer pain, they set the stage for the development of novel mechanism-based therapeutic approaches to treat these conditions.

## 3. Gene Therapy

A wide range of preclinical studies have been conducted to evaluate the efficacy of gene delivery to achieve meaningful benefit in experimental models with various levels of pain intensity. By modeling the nociceptive process that underlies pain generation and transmission, these studies help to determine the relative benefits of viral vector-based approaches for the attenuation of varying degrees of pain. Although many of the animal pain models were not developed to evaluate cancer-related nociception, nor do they provide a complete recapitulation of human conditions, they are meaningful for establishing potential therapeutics for cancer pain. As mentioned above, pain experienced by the cancer patient can be of a vast array. However, an understanding of these studies helps in providing scientific rationale for applying these approaches to cancer pain management. 

The use of viral vectors for the treatment of chronic pain involves the engineering of vector constructs in which the potentially harmful viral genomes have been replaced with nucleic acid sequences that encode a promoter to drive gene expression as well as an analgesic transgene. This approach exploits the inherent properties of the virus which allow for cell entry and transduction. In contrast to pharmacologic treatments, gene vectors allow for the persistent expression of therapeutic agents following a single administration of the viral vector rather than requiring repeated rounds of treatment. Vector delivery also evades the off-target side effects associated with systemic opioid use as well as avoiding the first pass response, which diminishes the activity of pharmacoagents before they reach the circulation. Furthermore, viral-based gene delivery represents an efficient method for gene delivery to various cells and tissues.

A suitable viral vector approach for treating cancer-related conditions would have to meet certain criteria, however. It would have to be safe and well tolerated. It should not elicit an immune response that would diminish the activity of the vector or the transgene nor would it exacerbate the pathological conditions of the cancer patient. The virus should also be replication incompetent rendering it incapable of producing an infection. Additionally, an ideal vector for gene transfer would be capable of infecting multiple cell types as well as expressing large and small transgenes. 

For the treatment of cancer pain, careful attention must also be paid to selecting a therapeutic gene that properly modulates the nociceptive cascade without causing additional complications to the patient. The transgene selected should be one capable of producing a gene product that has a known role in interfering with nociception. Such a gene might be involved in modulating neurotransmitter activity to regulate biochemical changes that underlie pain perception and transmission. The potential therapeutic agent should have known analgesic effects based on its association with a specific receptor or its ability to regulate changes in neural tissue that mediate pain pathogenesis. 

The efficacy of vector-derived therapeutic agents depends greatly on adequate gene transfer. Adequate gene expression, along with immune responses, remains the major limitations for gene transfer to the CNS. Therefore, the means of vector delivery must be carefully considered. This would involve choosing an optimal method to administer the therapy to the tissues involved in facilitating the pain syndrome. Possible approaches include targeting the neuroaxis by direct injection of sensory nerves, the spinal cord, or meninges, or the use of vectors with inherent or engineered tropisms for the dorsal root ganglia or other cells in the nociceptive circuitry.

### 3.1. Viral Vectors

A number of viral vectors are candidates for gene delivery. Vectors derived from adenovirus (Ad), adenoassociated virus (AAV), lentivirus, and herpes simplex virus (HSV) have been used as vehicles for transgene delivery to regulate pain using a number of laboratory pain models. These studies offer particular promise for cancer pain due to the scarcity of adequate cancer pain animal models. Therefore, a critical examination of gene therapy approaches used to modulate the nociceptive cascade in nonmalignant conditions is necessary to determine the best therapeutic strategy for cancer-associated pain conditions. Considering the complex nature of cancer pain, in that it does not have a singular source or origin, an understanding of successful gene therapy efforts to control pain originating from multiple sources can promote the use of the most appropriate palliative gene therapy method for treating cancer pain. 

#### 3.1.1. Adenoviral Vectors

 Adenoviral vectors are double-stranded, nonenveloped viral vectors that have been the most widely used for gene delivery [25]. Removal of the region encoding early genes impairs viral replication, rendering the vector suitable for gene transfer. Adenovirus can also be purified to high titers, and early gene expression can be achieved [25]. Furthermore, characterization of viral tropism has shown that it is capable of undergoing retrograde axonal transport [26–30]. No oncogenic properties have been associated with serotypes that have been used for gene therapy (serotypes 2 and 5) [31]. Moreover, adenovirus is a nonintegrating virus which eliminates the chance of its interfering with the normal function of endogenous genes [32]. Nevertheless, this also decreases the stability of the virus owing to its transient nature and short window of expression. Consequently, adenoviral vector-mediated gene delivery only provides transient expression. In addition, adenovirus tends to elicit a robust inflammatory response, making its application to therapy in eloquent neural structures problematic.

Adenoviral vectors have been used for the treatment of chronic pain. Using a rodent model of chronic pain induced by carrageenan injection into the plantar surface of the hind paw, Finegold et al. [33] were able to achieve a reduction in pain response at the spinal cord level. These studies helped to establish a CNS treatment paradigm for the treatment of chronic pain [33]. To do so, an adenoviral vector encoding a secreted form of the neuromodulatory peptide, beta-endorphin, was administered to the meninges surrounding the rat spinal cord, resulting in an attenuation of thermal hyperalgesia. Presumably beta-endorphin secretion from meningeal cells delivered the endogenous opioid to the spinal pain circuitry via direct spread into spinal cord tissue as well as into the cerebrospinal fluid in a fashion similar to epidural or intrathecal narcotic pumps. These studies were important because they, for the first time, demonstrated the ability of a viral vector-based approach to modulate pain transmission *in vivo*. 

These initial studies by Finegold et al. led to additional investigations into the ability of adenoviral vectors to transfer therapeutic genes to target specific areas of the CNS to modulate pain [33]. Independent studies conducted by Milligan et al. (2005) and Yao et al. (2003) using intrathecal delivery methods in rats have demonstrated the ability of adenoviral vector-driven expression of anti-inflammatory interleukin (IL)-10 or IL-2, respectively, to decrease pain phenotypes observed in sciatic nerve injury models of chronic pain [34, 35]. The antinociceptive properties of these immunoregulatory molecules had previously been demonstrated in studies involving direct protein injections into rodents [34, 36–39]. However, there was little to no justification for their clinical application. The short half-lives of IL-10 and IL-2 would require frequent readministration to a sensitive anatomical space resulting in insurmountable treatment-related costs. To increase the expression profiles of IL-10, Milligan et al. [34] generated an adenovirus expressing IL-10 and administered it intrathecally to animals that demonstrated thermal hyperalgesia and mechanical allodynia due to chronic constriction injury (CCI) of the sciatic nerve. Treated animals demonstrated decreased pain responses, lasting for up to three weeks postvector administration, compared to rats that had received sham surgeries [34]. In similar studies that investigated the benefits of adenovirus-driven expression of IL-2, Yao and colleagues [35] observed a decrease in sciatic nerve CCI pain response that was maintained for up to four weeks postvector administration. By using adenovirus to drive expression, these studies were able to prolong the expression of these anti-inflammatory cytokines, resulting in a sustained antinociceptive effect [34, 35]. 

GABA is a major inhibitory neurotransmitter that modulates afferent nociceptive transmission in the CNS and has been shown to exhibit antinociceptive properties [40–42]. Decreases in GABA expression induce nociceptive-like behaviors [40, 43]. Alternatively, GABA overexpression has been shown to attenuate the pain response. GABA is synthesized by glutamic acid decarboxylase (GAD), and exogenous induction of GAD can lead to increases in GABA production that inhibit pain due to spinal cord and peripheral nerve injury [44–46]. Interestingly, adenoviral gene delivery of GAD to the trigeminal ganglion was able to attenuate pain symptoms in an orofacial model of chronic pain in which formalin was injected into the upper lip and whisker pads of rats (*P* < .001) [42].

The relevance of neurotransmitter control for pain treatment is not limited to neuronal involvement. Astrocytes express factors that are important for neurotransmitter clearance from the synaptic cleft. The glial glutamate transporter (GLT-1) found in astrocytes has been shown to be important for glutamate uptake which is compromised in instances of neuropathic pain [47]. To understand the potential benefits of increased glutamate removal in conditions of chronic pain, an adenoviral vector was used to overexpress GLT-1 in the spinal cord of rats. Under inflammatory and neuropathic pain conditions, Ad-GLT1 was able to significantly reduce thermal hyperalgesia following carrageenan injection (*P* < .001) and prevent tactile allodynia following spinal nerve ligation (*P* < .001) [48]. These results suggest that Ad-driven GLT-1 overexpression in astrocytes attenuates the induction of pain.

#### 3.1.2. Adenoassociated Viral Vectors

Adenoviral vectors carry certain advantages such as ease of purification at high titers and an ability to drive extremely robust gene expression. However, these first generation viral vectors had a high incidence of immunogenicity. Thus, the inflammatory reaction to the vector could actually act as a source of pain. Consequently, there was a need to develop viral vectors that could overcome these pitfalls. The use of recombinant adenoassociated viral (AAV) vectors is currently employed. AAV has fundamental properties that make it an attractive gene therapy vector. AAV can also be introduced into multiple cell types and has long-term expression [49, 50]. Additionally, it has a decreased risk of immunogenicity due to the elimination of all viral sequences except the 145-base pair (bp) inverted terminal repeats (ITRs) [51]. Lastly, AAV has no etiologic association with any known diseases [51]. 

Many AAV serotypes have been discovered with distinct abilities to target select tissue types. Serotype 2 has been the most extensively studied form. As with any viral vector approach, the vector transduction efficiency depends on a number of factors. Tissue integrity, route of administration, animal age, dose and cellular immune response may determine how effectively the tissue is transduced [52, 53]. Moreover, the activity and efficiency of the promoters used to drive the gene of interest may also play a unique role in determining the expression profiles of the viral vectors [54]. 

Using AAV-driven gene expression in experimental pain models, several studies have demonstrated that stimulation of antinociceptive pathways in the spinal cord can decrease pain phenotypes in rats. Brain-derived neurotrophic factor (BDNF) has been shown to activate two major spinal cord systems that are important for regulating the neuroaxis: the descending modulatory serotonergic system and the inhibitory GABA system. AAV-mediated expression of BDNF in the spinal cord was able to permanently reverse allodynia and hyperalgesia caused by chronic constriction of the sciatic nerve [55]. The changes due to the activation of inhibitory systems were again seen using AAV vectors in studies involving GAD overexpression for GABA production. Kim et al. have demonstrated that AAV-GAD delivered to DRG neurons or the sciatic nerve can attenuate neuropathic pain induced by nerve transection, with sustained effects lasting more than 3 months [41, 56, 57]. These effects offered new insights into ways AAV-based approaches could be used to treat chronic pain.

A major transducer of excitatory transmission of the spinal cord dorsal horn is the N-methyl-D-aspartate (NMDA) receptor. This receptor has been suggested to be responsible for the development and maintenance of central sensitization [58]. Therefore, antagonism of NMDA receptors should allow for effective reduction of pain hypersensitivity following injury. Indeed, transgenic mice engineered to express an NMDA receptor 1 (NR1) flanked by loxp sites demonstrated a 70% decrease in formalin-induced paw pain following AAV-driven elimination of NR1 by Cre-mediated recombination [59]. The use of viral vectors to deliver Cre recombinase allowed from maximum NR1 reduction in localized areas of the CNS was considered to be critical for central sensitization initialization. NR1 elimination achieved by direct injections of AAV-Cre into the lumbar dorsal horn showed that spinally administered agents remain localized, providing a rationale for the use of CNS delivery of NR1 antagonists.

The targeting of spinal cord neurons, including both excitatory and inhibitory postsynaptic cells, carries the risk of unfavorable descending facilitation; however. Xu et al. (2003) evaded this condition by targeting dorsal root ganglia (DRG) neurons [60]. By using AAV for transgene overexpression of the *μ*-opioid receptor (*μ*-OR) in rats, researchers were able to achieve lasting increases in *μ*-OR expression (>6 months) marked by an enhancement of the antinociceptive effects of morphine following vector-opiate coadministration [60]. These results offer an alternative approach for the introduction of antinociceptive transgenes for the treatment of pain. By targeting presynaptic DRG neurons, basal nociceptive responses were preserved in either normal or inflammatory conditions, suggesting that the use of AAV-bearing opiate transgenes carries a substantially low risk of tolerance development. 

The idea of exploring alternative routes for vector administration was also demonstrated by Milligan et al. (2005) who used intrathecal delivery of AAV-IL-10 to not only prevent, but also reverse mechanical allodynia and thermal hyperalgesia in a rat model of neuropathic pain [61]. 

The difference in tropism of AAV serotypes is also determined by the route of injection. In particular, AAV6 has been shown to effectively transduce nociceptive neurons of the DRG following intrathecal delivery, a characteristic that has not been observed using AAV2 [62–64]. However, this increase in the vector efficiency of AAV6 is greatly impacted by the route of administration. Although intrathecal delivery of AAV6 expressing GFP results in a 60% increase in transgene expression in DRG neurons, relatively low levels of transduction were observed in these cells following sciatic nerve, intramuscular, subcutaneous, and systemic injections of the AAV6 vector [64]. This further emphasizes the need for careful selection of injection parameters when developing a therapy for chronic pain.

#### 3.1.3. Herpes Simplex Viral Vectors

The use of Herpes Simplex Viral (HSV) vectors also represents a viable approach to pain gene therapy. HSV is an enveloped, double-stranded naturally occurring virus that has evolved a retrograde transport mechanism and can effectively transduce sensory nerve terminals, DRG neurons, and the trigeminal ganglion [65]. Recombinant HSV-vectors have been engineered to be replication incompetent, yet retain the ability to undergo retrograde transport and infect cells of the nervous system [66–68]. These vectors have a relatively large cloning capacity allowing large genes, or alternatively, multiple genes to be inserted into the vector. HSV vectors can also be produced to clinically relevant titers to allow for application to large-scale human therapy. 

The inherent ability of HSV-vectors to infect the CNS and in particular DRG neurons makes it an attractive choice for gene-based pain control. The first description of the efficacy and feasibility of this approach was demonstrated by Wilson and colleagues [69] using an HSV vector to express opiate peptides in the rat spinal cord for the attenuation of acute pain. This resulted in an elimination of pain response lasting for at least 7 weeks posttreatment [69].

The coupling of HSV with opioid-associated peptides has been evaluated extensively since the early experiments of Wilson et al. (1999) [69]. HSV-mediated delivery of proenkephalin has been shown to have an analgesic effect in an inflammatory pain model of chronic pain, to be antinociceptive following topical administration in acute pain, to be antiallodynic in neuropathic pain, and to attenuate nociception in a model of bone cancer pain [23, 70–72]. This approach has also been taken in the brain. Indeed, HSV-proenkephalin injections into the amygdala reduced pain-like behaviors and delayed the second phase of the formalin test in rats [73]. 

As mentioned above, the GABAergic system is important for pain control. Selective loss of inhibitory synaptic current has been associated with pain development and maintenance. To circumvent these changes an HSV-based gene therapy approach was applied to an SNL model of neuropathic pain. HSV-bearing glutamic acid decarboxylase (GAD) was used to increase GABA synthesis, which reduced pain-like behaviors in rats [44]. 

Certain proinflammatory cytokines are released in the dorsal horn of the spinal cord in response to glia activation during neuropathic pain. Tumor necrosis factor-alpha (TNF-*α*) is among these molecules. To attenuate pain response in a rat model of spinal nerve ligation, HSV was used to drive the expression of the selective TNF-*α* antagonist, p55 sTNFR in DRG neurons [74]. This caused a reduction in nociceptive behaviors characteristic of mechanical allodynia and thermal hyperalgesia. Likewise, vector administration of the soluble TNF-*α* neutralizing receptor also decreased pain-like behaviors in a spinal cord injury pain model [75]. These results collectively highlight the benefits of suppressed TNF-*α* activity in the treatment of pain and the importance of modifying the inflammatory cascade in chronic pain conditions. Indirect approaches that increase the expression of anti-inflammatory cytokines are known to interfere with TNF-*α* expression. For example, increased expression of IL-4 with an HSV vector approach has been shown to produce antiallodynic effects. This is due to the ability of IL-4 to regulate TNF-*α* activity [76]. 

DRG-regulated control of nociceptive transmission has also been investigated using HSV-driven expression of neurotrophic factors known to reverse sensory abnormalities associated with neuropathic pain. Glial-derived neurotrophic factor (GDNF) is a member of the transforming growth factor-*β* superfamily that is abundantly expressed in the central and peripheral nervous systems. Increased GDNF expression has demonstrated a neuroprotective quality in a variety of models of neurodegenerative disease. These observations suggest that it has therapeutic value for a number of disorders such as Parkinson's disease, Amyotrophic lateral sclerosis, and stroke [77–79]. In relation to pain, intrathecal HSV-mediated GDNF overexpression has been shown to be antinociceptive and to reduce allodynia following subcutaneous administration in a rodent model of SNL pain. Nevertheless, these effects were short lived resulting in a subsequent decline 2.5 weeks after injection [80].

#### 3.1.4. Lentiviral Vectors

Lentiviral vectors are some of the most promising vectors for clinical application. Lentiviral vectors can achieve stable transgene expression, elicit a minimal inflammatory response, and infect both dividing and nondividing cells, including neurons [81, 82]. They can also achieve retrograde transport and are the most widely used vectors for gene transfer to the CNS. Most lentiviral vectors are based on human immunodeficiency virus type 1 (HIV-1). However, other types including the nonprimate equine infectious anemia virus (EIAV) and the feline immunodeficiency virus (FIV) have been employed [83–85]. 

Studies utilizing the HIV-1 and the EIAV virus for transgene expression of GDNF have shown that vector pretreatment into the spinal cord of adult rat results in a reduced pain response following SNL [86]. Robust transgene expression was observed in both neurons and glial cells. Lentiviral vectors also achieved partial reversal of thermal and mechanical allodynia, demonstrating that this vector approach was able to effectively modify conditions of neuropathic pain [86].

The functional relevance of modulating pain-inducing factors in glial cells was also evaluated using a sciatic nerve injury model of pain in rats. Not only is pain associated with altered neuronal function, but aberrant conditions in spinal glial cells can aid in activating the nociceptive signaling pathway. Nuclear factor-*κβ* (NF-*κβ*) has been shown to exacerbate pain by facilitating proinflammatory conditions in activated spinal glial cells [46, 87]. Lentivirus-directed expression of a selective NF-*κβ* suppressor into the spinal cord was shown to markedly reduce pain response following chronic nerve constriction injury as well as result in a downregulation of proinflammatory cytokines [88].

### 3.2. Gene Therapy Considerations

Despite remarkable advances in viral vector technology and the vast array of preclinical studies conducted to evaluate vector efficacy in a number of diseases, there are considerable challenges that still remain. One issue relates to viral vector targeting. In order for any viral vector to have therapeutic value it is necessary that measures be taken to ensure efficient transgene delivery to the appropriate cells with limited nontargeted effects. A lack of appropriate targeting could substantially hinder therapy efficacy. However, certain approaches have been taken to improve vector tropism. 

The use of cell type-specific promoters can increase appropriate localization of transgene as well as decrease the possibility of off-target cell infection. Moreover, approaches that involve the modification of cell attachment proteins have also been developed for lentiviral and AAV vectors [89, 90]. Specifically in AAV vector production, AAV2 vector genomes responsible for replication can be packaged with variant AAV capsids to create pseudotyped vectors. Due to the natural tropism of different AAV serotypes for certain tissues, these hybrid systems can allow for optimal targeting of a specific cell or tissue type. Moreover, the field has also evolved to the degree in which modifications can be made in capsid proteins to create chimeric vectors with increased insertion capacity and tropism [90]. Also, certain viruses have a natural propensity for tissue specificity as is the case with HSV vectors that inherently infect the CNS. Therefore, careful selection of the best promoter and vector system could allow for increased cell targeting. 

Increased cell targeting does not always lead to improved viral transduction efficiency, however. It has been shown that ubiquitination and degradation of capsids substantially limits their ability to reach the nucleus. To overcome this, AAV capsid domains subject to ubiquitination have been mutated to allow for increased vector stability, achieving up to a 100-fold increase in transgene expression [89, 91]. 

Cell-specific targeting and improved transduction are only a few concerns associated with viral vector use. Vector production costs often limit the translation of viral vector approaches to the clinical setting. Moreover, several rounds of clinical trials are often required to establish the optimal clinical protocol for maximum therapeutic benefit. Consideration of the route of administration (i.e., systemic versus localized treatment), dose, and the potential for toxic effects must also be evaluated when establishing a treatment protocol involving gene therapy approaches. If the advances are realized, viral vector-mediated transgene delivery could provide promise for a number of chronic conditions including cancer pain.

## 4. Cell Therapy

Cell transplantation therapy also appears to be a possible approach for the treatment of pain. Chromaffin cells naturally release substances shown to be effective in modulating pain transmission [92, 93]. These factors include GABA, galanin, serotonin, and met-enkephalin. Injection of chromaffin cells into the lumbar subarachnoid space reduces nociceptive behaviors in the formalin test (*P* < .05) [94]. Moreover, cell transplantation for pain has also been achieved using various cell lines that overexpress substances with known antinociceptive properties. Neuronal cells from a number of sources including human (hNT2.17 cell line) have been modified to secrete GABA, serotonin, galanin, or BDNF [95–100]. Transplantation of these cells results in reversal/reduction of pain-like behaviors in rodent models of chronic pain. Moreover, spinal cord injection of neural precursor cells induced to differentiate into a GABAergic phenotype is also suggested to be an effective strategy for reducing pain [101]. 

Glial cells (microglia, astrocytes, and oligodendrocytes) are found in the CNS and respond to changes that occur in the neuronal environment. They have been shown to engage in bidirectional communication with neurons to facilitate changes in the nervous system. One way these cells regulate conditions in the CNS is by expressing and interacting with chemical molecules that modulate the pain response to nerve injury. In particular, astrocytes have been shown to be activated in neuropathic pain conditions [102, 103]. To understand if astrocytes could be bioengineered to display a positive role in pain regulation, an inducible gene system was used to overexpress proenkephalin in astrocytes. Transfected cells were implanted into the subarachnoid space of rats. Mechanical and thermal hyperalgesia produced by chronic constriction of the sciatic nerve was evaluated. These studies found that the regulated release of the opioid peptide was able to attenuate neuropathic pain in rats [104]. The application of glial cell transplantation for the delivery of therapeutic molecules to the CNS was further emphasized using galanin secreting cells [105]. In these studies, immortalized galanin-overexpressing astrocytes were injected into the subarachnoid space of rats with chronic pain from sciatic nerve injury. Cell transplantation was able to effectively attenuate thermal hyperalgesia and mechanical allodynia for up to seven weeks after cell transplantation [105]. 

The benefits of stem cells and cells with stem cell-like properties have also been evaluated for the treatment of chronic pain. Embryonic stem cells transplanted into the spinal cord of rats that had spinal cord injuries not only reduced tissue damage, but also attenuated the pain response in both the formalin and von Frey hair tests [106]. Human mesenchymal stem cells (hMCs) have also been used to reduce pain symptoms in rodent pain models. Mice that received hMCs treatment to the lateral cerebral ventricle following sciatic nerve injury displayed reduced pain-like behaviors compared to controls [107].

## 5. Clinical Trials

Currently there is an on-going trial for cancer pain. Wolfe et al. began the first human gene therapy trial for cancer pain in 2008 [24, 108]. A phase 1 study based on preclinical studies that defined the benefits of a replication-defective HSV vector-bearing preproenkephalin will determine the safety of intradermal vector injections into patients with intractable cancer pain [23, 24, 108]. It is a dose escalating trial initiative that involves patients with moderate to severe malignant disease. Primary outcome measures of this study are adverse events and dose. Efficacy of the therapy will be assessed as a secondary measure determined by a numeric pain rating scale and the concurrent use of pain medications. A subsequent phase 2 trial will evaluate the benefits of HSV-preproenkephalin in patients with inflammatory pain. Individuals with chronic intractable focal pain from arthritis represent an ideal population to target [24]. Data obtained from these efforts hope to set the stage for clinical evaluations of the benefits of HSV-mediated GAD expression for the treatment of chronic pain. Preclinical studies have provided substantial evidence for the effectiveness of GAD overproduction in the attenuation of pain. Consequently, a clinical grade HSV-expressing GAD vector has been constructed and is currently being evaluated in a rodent model of neuropathic pain due to spinal nerve ligation [24]. If successful, Wolfe et al. hope to use this as support for an expedited clinical research initiative to evaluate HSV-GAD in a phase 2 trial in patients suffering from painful diabetic neuropathy [24]. Taken together, these research investigations could establish HSV gene therapy as a viable approach for the clinical treatment of chronic pain.

## 6. Emerging Strategies

Aberrant neural activity is a characteristic feature of conditions of chronic pain. A substantial population of cancer patients experience intractable cancer pain with varying etiologies. Careful consideration must be taken when developing therapies for this patient population. Cell transplantation therapies always carry concerns of tumorigenesis, graft rejection, cell migration, proliferation, and viability. Therapies developed using opiate peptides are only partially effective and may be subject to the same mechanisms of tolerance seen with pharmacological therapy. Regulation of neurotrophic factor expression, although shown to be beneficial in attenuating pain in laboratory models, cannot be assigned a strict antinociceptive function. Furthermore, it would be challenging to determine an effective dose for treatment. Activating or downregulating members of the immune system could have unexpected adverse events in that these cytokines and chemokines often play a role in multiple regulatory systems. Therefore, a targeted approach for local pain inhibition could have particular relevance for the treatment of cancer pain.

Clostridial neurotoxins are assuming substantial clinical relevance. The family of clostridial neurotoxins includes botulinum toxins (BoNT) and tetanus toxin (TeTx). Each protein consists of both a heavy and a light chain separated by a disulfide bond. These subunits dissociate following cell entry. The light chain contains the catalytic activity, whereas the heavy chain is responsible for cell internalization. Depending on the subtype, the light chain of clostridial neurotoxins cleaves one of the three SNARE proteins necessary for neurotransmitter release. Without the heavy chain, the light chain cannot achieve neuronal binding, yet its proteolytic activity remains intact. Therefore focal delivery of the light chain to target a select neuronal population is a viable approach for treating a number of conditions involving aberrant neuronal discharge. 

Several possible uses for clostridial toxins have been proposed. In relation to cancer pain, focal injections of BoNT have been used to reduce the intensity and frequency of pain due to leiomyomas (skin tumors) and have been proposed for the treatment of bone cancer pain [8, 109]. Support for BoNT translation to the cancer patient lies in its demonstrated ability to suppress key mediators of pain transmission and factors that have been hypothesized to regulate cancer nociception [8, 110, 111]. Such factors include substance P, endothelin-1, and calcitonin gene-related peptide (CGRP) [8, 9, 112]. Unfortunately, there are no known studies investigating the usefulness of BoNT gene transfer for the treatment of any condition. 

The benefits of gene transfer of TeTx light chain (TeTx.LC) in isolation of tumorigenesis and pain have been evaluated in our laboratory. Considering the common mode of action between BoNT and TeTx, these studies provide a basis for the exploration of gene-based neural inhibition using viral vectors expressing TeTx.LC. Using an adenovirus-based vector approach, we have demonstrated the ability of TeTx.LC to inhibit neural activity following direct injections into the spinal cord of rodents [113]. In these studies, adult rats received ipsilateral injections of Ad.TeTx.LC into the lumbar spinal cord. Hindlimb paralysis was used as an outcome measure of neuronal inhibition. At the peak of transgene expression we reported no loss of motor neurons, cell death, or adverse events related to vector administration. Moreover, due to the transient nature of adenoviral vectors, we were able to determine that motor functional changes induced by spinal cord injection of Ad.TeTx.LC were reversible, indicating a lack of reorganization of neural networks and neurotoxic effects. These studies offer compelling evidence that this vector-based approach could be used to modulate conditions of chronic pain involving excessive neuronal firing. Moreover, since this is a pathophysiological feature of cancer pain, these studies invite the investigation of viral vector delivery of TeTx.LC using protocols established for safe focal delivery of therapies to the CNS.

## 7. Conclusions

Gene therapy is an exciting prospect for the treatment and management of cancer-related pain. Advances in viral vector design and increased understanding of the signaling systems that underlie pain generation, transmission, and maintenance have helped to establish gene-based approaches as a potentially effective means to modulate nociception in a number of diseases. The application of these gene therapy tools using cancer pain laboratory models provides substantial insight into the molecular and biological features of virus-derived vectors under tumorigenic conditions. Animal work with state-of-the-art gene therapy technology demonstrates the feasibility of targeted gene expression of factors that play a known role in the nociceptive cascade. Considering the high prevalence of pain among the cancer patient population, the development of viral vector-based therapy techniques is timely and necessary. Moreover, since the etiology of the pain experienced by cancer patients can arise from a sensory neuropathy distinct from the cancer itself (i.e., therapy side effect or diagnostic and surgical procedures), a mechanistic approach to cancer-associated pain is crucial to improving the quality and survival of the cancer patient. Therefore, careful selection of transgene and vector system must be taken into consideration when designing studies aimed at addressing conditions of cancer pain. Clinical investigations to assess the safety, tolerability, and benefits of gene-based therapies promise to direct the field towards focal continuous delivery of analgesic peptides to selectively disrupt nociceptive signaling with limited off-target effects. Although some risk still remains, gene therapy-based pain management could prove to be a valuable interventional procedure that sets the stage for a new class of medicines which are effective at alleviating pain symptoms that are associated with a number of disorders.

## References

[1] Meuser T, Pietruck C, Radbruch L, Stute P, Lehmann KA, Grond S (2001). Symptoms during cancer pain treatment following WHO-guidelines: a longitudinal follow-up study of symptom prevalence, severity and etiology. *Pain*.

[2] Mantyh PW (2006). Cancer pain and its impact on diagnosis, survival and quality of life. *Nature Reviews Neuroscience*.

[3] Tolle SW, Tilden VP, Rosenfeld AG, Hickman SE (2000). Family reports of barriers to optimal care of the dying. *Nursing Research*.

[4] Christo PJ, Mazloomdoost D (2008). Cancer pain and analgesia. *Annals of the New York Academy of Sciences*.

[5] Dubner R, Gold M (1999). The neurobiology of pain. *Proceedings of the National Academy of Sciences of the United States of America*.

[6] Millan MJ (1999). The induction of pain: an integrative review. *Progress in Neurobiology*.

[7] Woolf CJ, Mannion RJ (1999). Neuropathic pain: aetiology, symptoms, mechanisms, and management. *Lancet*.

[8] Namazi H (2008). A novel use of botulinum toxin to ameliorate bone cancer pain. *Annals of Surgical Oncology*.

[9] Ahmed SI, Thompson J, Coulson JM, Woll PJ (2000). Studies on the expression of endothelin, its receptor subtypes, and converting enzymes in lung cancer and in human bronchial epithelium. *American Journal of Respiratory Cell and Molecular Biology*.

[10] Medhurst SJ, Walker K, Bowes M (2002). A rat model of bone cancer pain. *Pain*.

[11] Brown DC, Iadarola MJ, Perkowski SZ (2005). Physiologic and antinociceptive effects of intrathecal resiniferatoxin in a canine bone cancer model. *Anesthesiology*.

[12] Nagamine K, Ozaki N, Shinoda M (2006). Mechanical allodynia and thermal hyperalgesia induced by experimental squamous cell carcinoma of the lower gingiva in rats. *Journal of Pain*.

[13] Sevcik MA, Jonas BM, Lindsay TH (2006). Endogenous opioids inhibit early-stage pancreatic pain in a mouse model of pancreatic cancer. *Gastroenterology*.

[14] Tyner TR, Parks N, Faria S (2007). Effects of collagen nerve guide on neuroma formation and neuropathic pain in a rat model. *American Journal of Surgery*.

[15] Pacharinsak C, Beitz A (2008). Animal models of cancer pain. *Comparative Medicine*.

[16] Jimenez-Andrade JM, Mantyh WG, Bloom AP (2010). Bone cancer pain. *Annals of the New York Academy of Sciences*.

[17] Sevcik MA, Ghilardi JR, Peters CM (2005). Anti-NGF therapy profoundly reduces bone cancer pain and the accompanying increase in markers of peripheral and central sensitization. *Pain*.

[18] Halvorson KG, Kubota K, Sevcik MA (2005). A blocking antibody to nerve growth factor attenuates skeletal pain induced by prostate tumor cells growing in bone. *Cancer Research*.

[19] Regan JM, Peng P (2000). Neurophysiology of cancer pain. *Cancer Control*.

[20] Javery KB, Ussery TW, Steger HG, Colclough GW (1996). Comparison of morphine and morphine with ketamine for postoperative analgesia. *Canadian Journal of Anaesthesia*.

[21] Pud D, Eisenberg E, Spitzer A, Adler R, Fried G, Yarnitsky D (1998). The NMDA receptor antagonist amantadine reduces surgical neuropathic pain in cancer patients: a double blind, randomized, placebo controlled trial. *Pain*.

[22] McQuay H (1999). Opioids in pain management. *Lancet*.

[23] Goss JR, Harley CF, Mata M (2002). Herpes vector-mediated expression of proenkephalin reduces bone cancer pain. *Annals of Neurology*.

[24] Wolfe D, Mata M, Fink DJ (2009). A human trial of HSV-mediated gene transfer for the treatment of chronic pain. *Gene Therapy*.

[25] McConnell MJ, Imperiale MJ (2004). Biology of adenovirus and its use as a vector for gene therapy. *Human Gene Therapy*.

[26] Ghadge GD, Roos RP, Kang UJ (1995). CNS gene delivery by retrograde transport of recombinant replication-defective adenoviruses. *Gene Therapy*.

[27] Kuo H, Ingram DK, Crystal RG, Mastrangeli A (1995). Retrograde transfer of replication deficient recombinant adenovirus vector in the central nervous system for tracing studies. *Brain Research*.

[28] Boulis NM, Turner DE, Dice JA, Bhatia V, Feldman EL (1999). Characterization of adenoviral gene expression in spinal cord after remote vector delivery. *Neurosurgery*.

[29] Boulis NM, Turner DE, Imperiale MJ, Feldman EL (2002). Neuronal survival following remote adenovirus gene delivery. *Journal of Neurosurgery*.

[30] Boulis NM, Noordmans AJ, Song DK (2003). Adeno-associated viral vector gene expression in the adult rat spinal cord following remote vector delivery. *Neurobiology of Disease*.

[31] Bardell D (1983). Leakage of cellular enzyme during replication of oncogenic and nononcogenic adenoviruses. *Archives of Virology*.

[32] Mitani K, Kubo S (2002). Adenovirus as an integrating vector. *Current Gene Therapy*.

[33] Finegold AA, Mannes AJ, Iadarola MJ (1999). A paracrine paradigm for in vivo gene therapy in the central nervous system: treatment of chronic pain. *Human Gene Therapy*.

[34] Milligan ED, Langer SJ, Sloane EM (2005). Controlling pathological pain by adenovirally driven spinal production of the anti-inflammatory cytokine, interleukin-10. *European Journal of Neuroscience*.

[35] Yao MZ, Gu JF, Wang JH, Sun LY, Liu H, Liu XY (2003). Adenovirus-mediated interleukin-2 gene therapy of nociception. *Gene Therapy*.

[36] Jiang CL, Xu D, Lu CL, Wang YX, You ZD, Liu XY (2000). Interleukin-2: structural and biological relatedness to opioid peptides. *NeuroImmunomodulation*.

[37] Jiang CL, You ZD, Lu CL (2000). Leu-enkephalin induced by IL-2 administration mediates analgesic effect of IL-2. *NeuroReport*.

[38] Wang Y, Pei G, Cai YC (1997). Human interleukin-2 could bind to opioid receptor and induce corresponding signal transduction. *NeuroReport*.

[39] Lotze MT, Frana LW, Sharrow SO (1985). In vivo administration of purified human interleukin 2. I. Half-life and immunologic effects of the Jurkat cell line-derived interleukin 2. *Journal of Immunology*.

[40] Meisner JG, Marsh AD, Marsh DR (2010). Loss of GABAergic interneurons in laminae I-III of the spinal cord dorsal horn contributes to reduced GABAergic tone and neuropathic pain after spinal cord injury. *Journal of Neurotrauma*.

[41] Kim J, Yoon YS, Lee H, Chang JW (2008). AAV-GAD gene for rat models of neuropathic pain and Parkinson’s disease. *Acta Neurochirurgica*.

[42] Vit JP, Ohara PT, Sundberg C (2009). Adenovector GAD65 gene delivery into the rat trigeminal ganglion produces orofacial analgesia. *Molecular Pain*.

[43] Moore KA, Kohno T, Karchewski LA, Scholz J, Baba H, Woolf CJ (2002). Partial peripheral nerve injury promotes a selective loss of GABAergic inhibition in the superficial dorsal horn of the spinal cord. *Journal of Neuroscience*.

[44] Hao S, Mata M, Wolfe D, Glorioso JC, Fink DJ (2005). Gene transfer of glutamic acid decarboxylase reduces neuropathic pain. *Annals of Neurology*.

[45] Liu J, Wolfe D, Hao S (2004). Peripherally delivered glutamic acid decarboxylase gene therapy for spinal cord injury pain. *Molecular Therapy*.

[46] Lee KM, Kang BS, Lee HL (2004). Spinal NF-kB activation induces COX-2 upregulation and contributes to inflammatory pain hypersensitivity. *European Journal of Neuroscience*.

[47] Beart PM, O’Shea RD (2007). Transporters for L-glutamate: an update on their molecular pharmacology and pathological involvement. *British Journal of Pharmacology*.

[48] Maeda S, Kawamoto AI, Yatani Y, Shirakawa H, Nakagawa T, Kaneko S (2008). Gene transfer of GLT-1, a glial glutamate transporter, into the spinal cord by recombinant adenovirus attenuates inflammatory and neuropathic pain in rats. *Molecular Pain*.

[49] Lai CM, Lai YKY, Rakoczy PE (2002). Adenovirus and adeno-associated virus vectors. *DNA and Cell Biology*.

[50] McCarty DM, Monahan PE, Samulski RJ (2001). Self-complementary recombinant adeno-associated virus (scAAV) vectors promote efficient transduction independently of DNA synthesis. *Gene Therapy*.

[51] Thomas CE, Ehrhardt A, Kay MA (2003). Progress and problems with the use of viral vectors for gene therapy. *Nature Reviews Genetics*.

[52] Kwon I, Schaffer DV (2008). Designer gene delivery vectors: molecular engineering and evolution of adeno-associated viral vectors for enhanced gene transfer. *Pharmaceutical Research*.

[53] Bostick B, Ghosh A, Yue Y, Long C, Duan D (2007). Systemic AAV-9 transduction in mice is influenced by animal age but not by the route of administration. *Gene Therapy*.

[54] Gregorevic P, Blankinship MJ, Allen JM (2004). Systemic delivery of genes to striated muscles using adeno-associated viral vectors. *Nature Medicine*.

[55] Eaton MJ, Blits B, Ruitenberg MJ, Verhaagen J, Oudega M (2002). Amelioration of chronic neuropathic pain after partial nerve injury by adeno-associated viral (AAV) vector-mediated over-expression of BDNF in the rat spinal cord. *Gene Therapy*.

[56] Kim J, Kim SJ, Lee H, Chang JW (2009). Effective neuropathic pain relief through sciatic nerve administration of GAD65-expressing rAAV2. *Biochemical and Biophysical Research Communications*.

[57] Kim SJ, Lee WI, Lee YS (2009). Effective relief of neuropathic pain by adeno-associated virus-mediated expression of a small hairpin RNA against GTP cyclohydrolase 1. *Molecular Pain*.

[58] Woolf CJ, Salter MW (2000). Neuronal plasticity: increasing the gain in pain. *Science*.

[59] South SM, Kohno T, Kaspar BK (2003). A conditional deletion of the NR1 subunit of the NMDA receptor in adult spinal cord dorsal horn reduces NMDA currents and injury-induced pain. *Journal of Neuroscience*.

[60] Xu Y, Gu Y, Xu GY, Wu P, Li GW, Huang LYM (2003). Adeno-associated viral transfer of opioid receptor gene to primary sensory neurons: a strategy to increase opioid antinociception. *Proceedings of the National Academy of Sciences of the United States of America*.

[61] Milligan ED, Sloane EM, Langer SJ (2005). Controlling neurophatic pain by adeno-associated virus driven production of the anti-inflammatory cytokine, interleukin-10. *Molecular Pain*.

[62] Xu Y, Gu Y, Wu P, Li GW, Huang LYM (2003). Efficiencies of transgene expression in nociceptive neurons through different routes of delivery of adeno-associated viral vectors. *Human Gene Therapy*.

[63] Storek B, Harder NM, Banck MS (2006). Intrathecal long-term gene expression by self-complementary adeno-associated virus type I suitable for chronic pain studies in rats. *Molecular Pain*.

[64] Towne C, Pertin M, Beggah AT (2009). Recombinant adeno-associated virus serotype 6 (rAAV2/6)-mediated gene transfer to nociceptive neurons through different routes of delivery. *Molecular Pain*.

[65] Frampton AR, Goins WF, Nakano K, Burton EA, Glorioso JC (2005). HSV trafficking and development of gene therapy vectors with applications in the nervous system. *Gene Therapy*.

[66] Maguire-Zeiss KA, Bowers WJ, Federoff HJ (2001). HSV vector-mediated gene delivery to the central nervous system. *Current Opinion in Molecular Therapeutics*.

[67] Lilley CE, Groutsi F, Han Z (2001). Multiple immediate-early gene-deficient herpes simplex virus vectors allowing efficient gene delivery to neurons in culture and widespread gene delivery to the central nervous system in vivo. *Journal of Virology*.

[68] Fink DJ, Sternberg LR, Weber PC, Mata M, Goins WF, Glorioso JC (1992). In vivo expression of *β*-galactosidase in hippocampal neurons by HSV-mediated gene transfer. *Human Gene Therapy*.

[69] Wilson SP, Yeomans DC, Bender MA, Lu Y, Goins WF, Glorioso JC (1999). Antihyperalgesic effects of infection with a preproenkephalin-encoding herpes virus. *Proceedings of the National Academy of Sciences of the United States of America*.

[70] Hao S, Mata M, Goins W, Glorioso JC, Fink DJ (2003). Transgene-mediated enkephalin release enhances the effect of morphine and evades tolerance to produce a sustained antiallodynic effect in neuropathic pain. *Pain*.

[71] Goss JR, Mata M, Goins WF, Wu HH, Glorioso JC, Fink DJ (2001). Antinociceptive effect of a genomic herpes simplex virus-based vector expressing human proenkephalin in rat dorsal root ganglion. *Gene Therapy*.

[72] Yeomans DC, Lu Y, Laurito CE (2006). Recombinant herpes vector-mediated analgesia in a primate model of hyperalgesia. *Molecular Therapy*.

[73] Kang W, Wilson MA, Bender MA, Glorioso JC, Wilson SP (1998). Herpes virus-mediated preproenkephalin gene transfer to the amygdala is antinociceptive. *Brain Research*.

[74] Hao S, Mata M, Glorioso JC, Fink DJ (2007). Gene transfer to interfere with TNF*α* signaling in neuropathic pain. *Gene Therapy*.

[75] Peng XM, Zhou ZG, Glorioso JC, Fink DJ, Mata M (2006). Tumor necrosis factor-*α* contributes to below-level neuropathic pain after spinal cord injury. *Annals of Neurology*.

[76] Hao S, Mata M, Glorioso JC, Fink DJ (2006). HSV-mediated expression of interleukin-4 in dorsal root ganglion neurons reduces neuropathic pain. *Molecular Pain*.

[77] Bohn MC, Connor B, Kozlowski DA, Mohajeri MH (2000). Gene transfer for neuroprotection in animal models of Parkinson’s disease and amyotrophic lateral sclerosis. *Novartis Foundation Symposium*.

[78] Chen Q, He YI, Yang K (2005). Gene therapy for Parkinson’s disease: progress and challenges. *Current Gene Therapy*.

[79] Wang Y, Alexander OB, Woodward-Pu YM, Stahl CE, Borlongan CV (2006). Viral vector strategy for glial cell line-derived neurotrophic factor therapy for stroke. *Frontiers in Bioscience*.

[80] Hao S, Mata M, Wolfe D, Huang S, Glorioso JC, Fink DJ (2003). HSV-mediated gene transfer of the glial cell-derived neurotrophic factor provides an antiallodynic effect on neuropathic pain. *Molecular Therapy*.

[81] Escors D, Breckpot K (2010). Lentiviral vectors in gene therapy: their current status and future potential. *Archivum Immunologiae et Therapiae Experimentalis*.

[82] Naldini L, Blomer U, Gage FH, Trono D, Verma IM (1996). Efficient transfer, integration, and sustained long-term expression of the transgene in adult rat brains injected with a lentiviral vector. *Proceedings of the National Academy of Sciences of the United States of America*.

[83] Poeschla EM (2003). Non-primate lentiviral vectors. *Current Opinion in Molecular Therapeutics*.

[84] Saenz DT, Poeschla EM (2004). FIV: from lentivirus to lentivector. *Journal of Gene Medicine*.

[85] Azzouz M, Mazarakis N (2004). Non-primate EIAV-based lentiviral vectors as gene delivery system for motor neuron diseases. *Current Gene Therapy*.

[86] Pezet S, Krzyzanowska A, Wong LF (2006). Reversal of neurochemical changes and pain-related behavior in a model of neuropathic pain using modified lentiviral vectors expressing GDNF. *Molecular Therapy*.

[87] Laughlin TM, Bethea JR, Yezierski RP, Wilcox GL (2000). Cytokine involvement in dynorphin-induced allodynia. *Pain*.

[88] Meunier A, Latrémolière A, Dominguez E (2007). Lentiviral-mediated targeted NF-*κ*B blockade in dorsal spinal cord glia attenuates sciatic nerve injury-induced neuropathic pain in the rat. *Molecular Therapy*.

[89] Heilbronn R, Weger S (2010). Viral vectors for gene transfer: current status of gene therapeutics. *Handbook of Experimental Pharmacology*.

[90] Waehler R, Russell SJ, Curiel DT (2007). Engineering targeted viral vectors for gene therapy. *Nature Reviews Genetics*.

[91] Zhong L, Li B, Mah CS (2008). Next generation of adeno-associated virus 2 vectors: point mutations in tyrosines lead to high-efficiency transduction at lower doses. *Proceedings of the National Academy of Sciences of the United States of America*.

[92] Wilson SP, Chang KJ, Viveros OH (1981). Opioid peptide synthesis in bovine and human adrenal chromaffin cells. *Peptides*.

[93] Unsicker K (1993). The trophic cocktail made by adrenal chromaffin cells. *Experimental Neurology*.

[94] Siegan JB, Sagen J (1997). Attenuation of formalin pain responses in the rat by adrenal medullary transplants in the spinal subarachnoid space. *Pain*.

[95] Eaton MJ, Wolfe SQ, Martinez M (2007). Subarachnoid transplant of a human neuronal cell line attenuates chronic allodynia and hyperalgesia after excitotoxic spinal cord injury in the rat. *Journal of Pain*.

[96] Eaton MJ, Karmally S, Martinez MA, Plunkett JA, Lopez T, Cejas PJ (1999). Lumbar transplant of neurons genetically modified to secrete galanin reverse pain-like behaviors after partial sciatic nerve injury. *Journal of the Peripheral Nervous System*.

[97] Eaton MJ, Plunkett JA, Martinez MA (1999). Transplants of neuronal cells bioengineered to synthesize GABA alleviate chronic neuropathic pain. *Cell Transplantation*.

[98] Eaton MJ, Santiago DI, Dancausse HA, Whittemore SR (1997). Lumbar transplants of immortalized serotonergic neurons alleviate chronic neuropathic pain. *Pain*.

[99] Cejas PJ, Martinez M, Karmally S (2000). Lumbar transplant of neurons genetically modified to secrete brain-derived neurotrophic factor attenuates allodynia and hyperalgesia after sciatic nerve constriction. *Pain*.

[100] Eaton MJ, Wolfe SQ (2009). Clinical feasibility for cell therapy using human neuronal cell line to treat neuropathic behavioral hypersensitivity following spinal cord injury in rats. *Journal of Rehabilitation Research and Development*.

[101] Mukhida K, Mendez I, McLeod M (2007). Spinal GABAergic transplants attenuate mechanical allodynia in a rat model of neuropathic pain. *Stem Cells*.

[102] Garrison CJ, Dougherty PM, Kajander KC, Carlton SM (1991). Staining of glial fibrillary acidic protein (GFAP) in lumbar spinal cord increases following a sciatic nerve constriction injury. *Brain Research*.

[103] Ji RR, Kawasaki Y, Zhuang ZY, Wen YR, Decosterd I (2006). Possible role of spinal astrocytes in maintaining chronic pain sensitization: review of current evidence with focus on bFGF/JNK pathway. *Neuron Glia Biology*.

[104] Xu Y, Tian XB, An K, Yang H, Tian YUK (2008). Lumbar transplantation of immortalized enkephalin-expressing astrocytes attenuates chronic neuropathic pain. *European Journal of Pain*.

[105] An K, Xu Y, Yang H, Shu HH, Xiang HB, Tian YK (2010). Subarachnoid transplantation of immortalized galanin-overexpressing astrocytes attenuates chronic neuropathic pain. *European Journal of Pain*.

[106] Hendricks WA, Pak ES, Owensby JP (2006). Predifferentiated embryonic stem cells prevent chronic pain behaviors and restore sensory function following spinal injury in mice. *Molecular Medicine*.

[107] Siniscalco D, Giordano C, Galderisi U (2010). Intra-brain microinjection of human mesenchymal stem cells decreases allodynia in neuropathic mice. *Cellular and Molecular Life Sciences*.

[108] Wolfe D, Wechuck J, Krisky D, Mata M, Fink DJ (2009). A clinical trial of gene therapy for chronic pain. *Pain Medicine*.

[109] Onder M, Adişen E (2009). A new indication of botulinum toxin: leiomyoma-related pain. *Journal of the American Academy of Dermatology*.

[110] Singh R, Wang B, Shirvaikar A (1999). The IL-1 receptor and rho directly associate to drive cell activation in inflammation. *Journal of Clinical Investigation*.

[111] Dolly JO, Aoki KR (2006). The structure and mode of action of different botulinum toxins. *European Journal of Neurology*.

[112] Alanen K, Deng DX, Chakrabarti S (2000). Augmented expression of endothelin-1, endothelin-3 and the endothelin-B receptor in breast carcinoma. *Histopathology*.

[113] Teng Q, Tanase DK, Liu JK, Garrity-Moses ME, Baker KB, Boulis NM (2005). Adenoviral clostridial light chain gene-based synaptic inhibition through neuronal synaptobrevin elimination. *Gene Therapy*.

